# The emerging power and promise of non-coding RNAs in chronic pain

**DOI:** 10.3389/fnmol.2022.1037929

**Published:** 2022-11-03

**Authors:** Changteng Zhang, Rui Gao, Ruihao Zhou, Hai Chen, Changliang Liu, Tao Zhu, Chan Chen

**Affiliations:** ^1^Department of Anesthesiology and National Clinical Research Center for Geriatrics, West China Hospital, Sichuan University and The Research Units of West China (2018RU012), Chinese Academy of Medical Sciences, Chengdu, China; ^2^Laboratory of Anesthesia and Critical Care Medicine, National-Local Joint Engineering Research Centre of Translational Medicine of Anesthesiology, West China Hospital, Sichuan University, Chengdu, China; ^3^Department of Respiratory and Critical Care Medicine, West China Medical School/West China Hospital, Sichuan University, Chengdu, China; ^4^Department of Respiratory and Critical Care Medicine, Targeted Tracer Research and Development Laboratory, West China Hospital, Sichuan University, Chengdu, Sichuan, China

**Keywords:** non-coding RNA, microRNA, long non-coding RNA, circular RNA, chronic pain, biomarker

## Abstract

Chronic pain (CP) is an unpleasant sensory and emotional experience associated with, or resembling that associated with, actual or potential tissue damage lasting longer than 3 months. CP is the main reason why people seek medical care and exerts an enormous economic burden. Genome-wide expression analysis has revealed that diverse essential genetic elements are altered in CP patients. Although many possible mechanisms of CP have been revealed, we are still unable to meet all the analgesic needs of patients. In recent years, non-coding RNAs (ncRNAs) have been shown to play essential roles in peripheral neuropathy and axon regeneration, which is associated with CP occurrence and development. Multiple key ncRNAs have been identified in animal models of CP, such as microRNA-30c-5p, ciRS-7, and lncRNA MRAK009713. This review highlights different kinds of ncRNAs in the regulation of CP, which provides a more comprehensive understanding of the pathogenesis of the disease. It mainly focuses on the contributions of miRNAs, circRNAs, and lncRNAs to CP, specifically peripheral neuropathic pain (NP), diabetic NP, central NP associated with spinal cord injury, complex regional pain syndrome, inflammatory pain, and cancer-induced pain. In addition, we summarize some potential ncRNAs as novel biomarkers for CP and its complications. With an in-depth understanding of the mechanism of CP, ncRNAs may provide novel insight into CP and could become new therapeutic targets in the future.

## Introduction and chronic pain overview

Chronic pain (CP) is an unpleasant sensory and emotional experience associated with, or resembling that associated with, actual or potential tissue damage lasting longer than 3 months (Treede et al., 2015; Raja et al., 2020). Approximately 20% adults suffer pain and another 10% adults are diagnosed with CP worldwide each year (Goldberg and McGee, 2011). Patients with CP are often characterized as having allodynia (innocuous stimulus causing pain), hyperalgesia (noxious stimulus triggering an amplified response), and spontaneous pain (Woolf and Salter, 2000). Several factors are associated with the severity of CP, mainly including age, gender, genetics, ethnicity, cultural background, smoking, alcohol, physical activity, mental health, surgical and medical interventions, and even other attitudes and beliefs about pain, etc. (Mills et al., 2019). Moreover, comorbidities and complications are two difficult problems of CP. Patients with CP often have an increased co-occurrence of depression, cardiovascular disease, clinical insomnia, etc. (Jank et al., 2017; Macfarlane et al., 2017; van Hecke et al., 2017). Several common types of CP are summarized in this review, such as neuropathic pain (NP), complex regional pain syndrome (CRPS), inflammatory pain (IP), and cancer-induced pain (CIP) (Treede et al., 2019). Thereinto, the etiology of chronic NP can be divided into peripheral nerve lesions [such as peripheral nerve injury (PNI)-induced NP, diabetic NP (DNP), etc.] and central nerve lesions [such as spinal cord injury (SCI)] (Treede et al., 2019).

**FIGURE 1:**
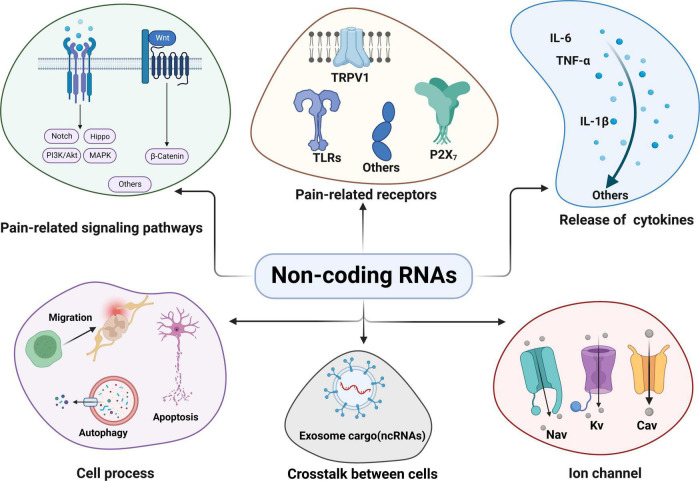
Non-coding RNAs (ncRNAs) and their regulatory mechanism in chronic pain: ncRNAs can participate in pain regulation through various mechanisms. Some common and key mechanisms mainly include pain-related signaling pathways; pain-related receptors and targets; regulation of cytokines release; some cell processes (cell migration, autophagy and apoptosis etc.); exosomal ncRNAs crosstalk between cells and ncRNAs targeting ion channels related to chronic pain. Akt, V-akt murine thymoma viral oncogene homolog; Cav, voltage-gated calcium; IL, interleukin; MAPK, mitogen-activated protein kinases; Kv, voltage-gated potassium channel; Nav, voltage-dependent sodium; ncRNAs, Non-coding RNAs; PI3K, phosphatidylinositol-3-kinase; TLRs, toll-like receptors; TNF, tumor necrosis factor; TRPV1, transient receptor potential vanilloid 1.

The mechanisms underlying CP have been explored for several decades, mainly including central sensitization, neuroinflammation, oxidative stress, etc. (Ji et al., 2018; Kaushik et al., 2020). However, the pathogenesis of CP has not been completely elucidated. Although several analgesic treatments are available, they are often hampered by side effects or limited efficacy (Sisignano et al., 2019). Elucidating the pathogenic mechanisms of CP may help seek novel specific biomarkers and efficient therapies for controlling the symptoms of CP. Notably, non-coding RNAs (ncRNAs) are altered greatly in both clinical research and pre-clinical models of CP and constitute a regulatory transcriptome network in the pathogenesis of CP (Ciszek et al., 2015; Peng et al., 2017; Du et al., 2022). Multiple ncRNAs are differentially expressed in the animal models of CP (Li et al., 2021b) and are strongly related to various mechanisms, such as pain-related signaling pathways, receptors, cytokines, cell processes, ion channels, and exosomes in cell-to-cell communications (Wang Z. et al., 2018; Xiang et al., 2019; Ma et al., 2021; Felix et al., 2022; Liu et al., 2022; Figure 1).

This review aims to summarize the recent evidence about the potential role of ncRNAs in CP. Understanding their functions may help to reveal the complex pathogenic mechanisms in CP and identify new potential diagnostic biomarkers and therapeutic targets.

### General aspects of non-coding RNAs

**FIGURE 2:**
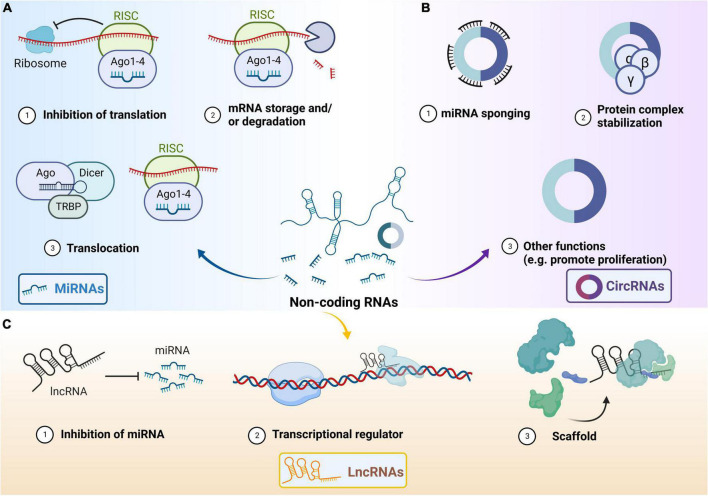
Main functions of three types of non-coding RNAs. **(A)** General functions of microRNA (miRNA). Unwinding of the miRNA *via* Argonaute, and transactivation response RNA-binding protein (TRBP)-dependent loading into the RNA-induced silencing complex (RISC). Binding of target mRNAs to miRNAs in RISC can result in translation repression or degradation of the mRNA. MiRNA and RISC can translocate to other cells. The RISC contains RNA-binding proteins including protein kinase RNA activator, TRBP and Dicer. **(B)** General functions of circular RNA (circRNA). CircRNA can act as miRNA sponge and protein complex stabilization. CircRNAs have other functions (including promoting cell proliferation etc.). **(C)** General functions of Long non-coding RNA (lncRNA). LncRNA can inhibit miRNA and act as transcriptional regulator. LncRNA participates in scaffolding complexes. The lncRNA possesses different domains that bind distinct effector molecules. The lncRNA can bind its multiple effector partners at the same time, which may have transcriptional activating or repressive activities, together in both time and space. Ago, Argonaute; circRNAs, circular RNAs; lncRNAs, long non-coding RNAs; miRNAs, microRNAs; RISC, RNA-induced silencing complex; TRBP, transactivation response RNA-binding protein.

Non-coding RNAs are defined as RNAs that are unable to translate into proteins (Beermann et al., 2016). Generally, ncRNAs are divided into two categories according to their sequence length: (I) small ncRNAs (few to 200 nt), including microRNA (miRNA), ribosomal, small nuclear, piwi-interacting, and small interfering RNA; and (II) long ncRNA (lncRNA) (longer than 200 nt) (Beermann et al., 2016; Hombach and Kretz, 2016). We mainly introduce some general aspects of the three common types of ncRNAs: miRNA, circular RNA (circRNA), and lncRNA as follows (Esteller, 2011; Westholm and Lai, 2011; Kristensen et al., 2019; Figure 2).

MicroRNAs, endogenous and single-stranded RNAs ranging between 21 and 25 nucleotides in length, widely regulate gene expression, and more than 60% of genes are targets of miRNAs (Friedman et al., 2009). MiRNA has uridine at its 5′-end and is partially complementary to the 3′-end untranslated regions of the messenger RNA (mRNA). MiRNAs are produced by two RNase III proteins, Drosha and Dicer (Vishnoi and Rani, 2017). Pre-miRNAs are cleaved and processed by Drosha in the nucleus and guided to the cytoplasm (Ma et al., 2008; Westholm and Lai, 2011). Then, the pre-miRNA unwinds and one strand is incorporated into the RNA-induced silencing complex which is composed of Dicer, the double-stranded RNA-binding protein TRBP, and Argonaute2 proteins, ultimately resulting in translation repression or degradation of the mRNA (Gregory et al., 2005; Wu et al., 2006). The critical roles of miRNAs in animal models of CP, such as the severe dysregulation of miRNAs in the dorsal root ganglion (DRG) and the spinal cord regions, have been reported in many studies (Bali et al., 2013; Peng et al., 2017; Sakai et al., 2017).

Circular RNAs are generally produced through back-splicing or exon skipping of pre-mRNAs, while a single genomic location can generate multiple types of circRNAs. CircRNAs are notable for their continuous closed-loop structure, named a “back-splicing” structure, which is primarily formed through the junction of a downstream 3′ splice site with an upstream 5′ splice site (Zhang et al., 2016). Due to these structures, circRNAs are abnormally stable for exonucleases and are widely involved in various life processes such as cell proliferation, differentiation, and apoptosis (Haque and Harries, 2017). CircRNAs play key roles in mRNA stability and function through their activities as miRNA sponges, translation modulators, protein complex stabilization, and biomarkers (Hansen et al., 2013). CircRNAs are stable in the human bloodstream under physiological or pathological conditions and may be a potential biomarker easily obtained for clinical CP patients in the future (Vea et al., 2018).

Long non-coding RNAs have more than 200 nucleotides and can interact with proteins, DNAs, and multiple RNAs (Ulitsky and Bartel, 2013). LncRNAs lack protein-coding potential and often harbor a poly-A tail and can be spliced, which has been shown to play vital roles in a variety of biological processes such as translational inhibition, transcriptional silencing, promoter-specific gene regulation, X-chromosome inactivation, imprinting, maintenance of nuclear architecture and modulation of multiprotein complexes (Lee, 2009; Ponting et al., 2009; Statello et al., 2021). An estimated abundance of 5,400 to more than 10,000 lncRNA transcripts has been reported in humans (Jia et al., 2010; Djebali et al., 2012; Lorenzi et al., 2021) and several studies have shown that lncRNAs contribute to the occurrence of CP in some pre-clinical models by targeting pain-associated genes or other downstream molecules and increasing neuronal excitability in DRG primary sensory neurons (Zhao et al., 2013; Du et al., 2022).

Several factors are associated with expression of ncRNA. Current evidence has demonstrated that common traits, including age, sex, smoking, body mass, and physical activity, can influence circulating small ncRNA expression levels (Rounge et al., 2018), among which aging is a strong factor. For example, a previous study has analyzed miRNAs from the whole blood of 5,221 individuals and found that 127 of 150 miRNAs are affected by age (Huan et al., 2018). In addition, different exercise training programs greatly alter ncRNAs. Researchers have identified 204 differentially expressed lncRNAs (DELs) from untrained young individuals after 12 weeks of high-intensity interval training, whereas 43 DELs are identified after resistance training (Bonilauri and Dallagiovanna, 2020).

In this review, we provide an overview of the recent research progress and specific roles of ncRNAs in CP and focus on the alterations of ncRNAs reported mainly in the blood samples, DRG, spinal cord dorsal horn (SDH), and injured peripheral nerves of animal models and patients suffering CP.

### Non-coding RNAs: Insights into the molecular mechanism of chronic pain

#### Peripheral neuropathic pain-associated with peripheral nerve injury

Neuropathic pain affects 8% of adults worldwide and is commonly caused by somatosensory system lesions or diseases, such as PNI (Colloca et al., 2017; Finnerup et al., 2021). PNI is a major onset cause of NP in adults, characterized by allodynia and pain hypersensitivity, and accompanied by ncRNAs alterations in the DRG or peripheral blood in patients (Arthur-Farraj et al., 2017; Hu et al., 2021). An increasing number of researchers have focused on the potential of ncRNAs as biomarkers in PNI-induced NP (Table 1).

**TABLE 1:** Non-coding RNAs in periphery neuropathic pain (including PNI and DNP).

Classification	Model	ncRNAs	Expression	Tissue	Target	PMID
MiRNA	SNI	miR-30c-5p	Up	SC, DRG, CSF, and plasma	TGF-β	30089634
	SNL	miR-143	Down	DRG	Dnmt3a	29170626
		miR-21	Up	DRG	TLR8	30455267
		miR-124-3p	Down	DRG/SDH	Egr1	34008206
	CCI	miR-206-3p	Down	DRG	HDAC4	30587671
		miR-223	Down	SC	NLRP3/IL-1β	34408803
		MiR-144	Down	DRG	RASA1	31737949
		miR-140-3p	Down	DRG	JAG1	34269482
		miR-122-5p	Down	SC	PDK4	33566299
		miR-30b-5p	Down	SC	CYP24A1-Wnt/beta-catenin signaling	34748047
		miR-340-5p	Down	SC	Rap1A	30848438
		miRNA-146a-5p	Up	DRG and SDH	IRAK1/TRAF6	29885668
	DNP	miR-190a-5p	Down	SC	SLC17A6	29042815
		miR-155	Up	SC	Nrf2	31404910
		miR-193a	Down	Lumbar SDH	HMGB1	31665716
		miR-590-3p	Down	DRG	RAP1A	33332780
		miR-133a-3p	Up	SN	p38MAPK	32632603
CircRNA	SNI	circRNA.2837	Down	SN	miR-34 family members	30098504
	SCiatic nerve crush model	circAnkib1	Down	SN	miR-423-5p, miR-485-5p, miR-666-3p/Cyp26b1	31415184
	CCI	CIRCZNF609	Unknown	Unknown	miR-22-3p/ENO1	32827683
		circ_0005075	Up	DRG	miR-151a-3p/NOTCH2	32860901
		ciRS-7	Up	SDH	miR-135a-5p	31978512
		circZRANB1	Down	SC	miR-24-3p/LPAR3	32777532
		circRNA_013779	Up	SDH	Unknown	28761373
		circRNA_008008	Up	SDH	Unknown	
		circRNA_003724	Up	SDH	Unknown	
	DNP	circHIPK3	Up	DRG	miR-124	30286957
		mmu_circ_0010794	Up	SC	Unknown	34055074
		mmu_circ_0006623	Up	SC	Unknown	
		mmu_circ_0006175	Up	SC	Unknown	
		mmu_circ_0016083	Down	SC	Unknown	
		mmu_circ_0006471	Down	SC	Unknown	
LncRNA	SNI	lncRNA DGCR5	Down	SC	miR-330-3p/PDCD4	30317600
		lncRNA LOC100911498	Up	SC	P2X_4_R, BDNF, p38	33949153
		lncRNA Linc01119	Up	SC and DRG	ELAVL1/BDNF	34234645
	SNL	lncRNA SNHG1	Up	SC	CDK4	33336721
		lncRNA SNHG4	Up	SC	miR-423-5p	32454787
		lncRNA SNHG5	Up	DRG	miR-154-5p/CXCL13	32248399
		lncRNA H19	Up	DRG	Unknown	32099850
		lncRNA PKIA-AS1	Up	SC	CDK6	30873006
		Linc00052	Up	SC	miR-448/JAK1	32012267
		lncRNA Lncenc1	Up	DRG	EZH2/BAI1	33340495
	CCI	lncRNA FIRRE	Up	SC	HMGB1	33151463
		lncRNA XIST	Up	SC	miR-154-5p/TLR5	30335888
			Up	SC	miR-150/ZEB1	29323698
			Up	SC	miR-544/STAT3	29219175
		lncRNA MALAT1	Up	SC	miR-129-5p/HMGB1	32065547
			Up	SC	miR-154-5p/AQP9	31746418
		lncRNA CRNDE	Up	SC	miR-136/IL-6R	34612146
		lncRNA DILC	Up	SC	SOCS3/JAK2/STAT3	32510145
		lncRNA uc.153	Up	SC	miR-182-5p/EphB1-NMDA	32701835
		LncRNA NEAT1	Up	SC	miR-381/HMGB1	29633273
		lncRNA SNHG16	Up	SC	miR-124-3p, miR-141-3p/JAG1	33049352
		lncRNA GAS5	Down	SC	miR-452-5p/CELF2	33264082
		lncRNA MRAK009713	Up	DRG	P2X_3_	28708759
	DNP	lncRNA NON-RATT021972	Up	DRG	P2X_7_	28928602
		lncRNA uc.48+	Up	DRG	P2X_3_	26686228
		lncRNA BC168687	Up	DRG	P2X_7_	29204447
			Up	DRG	TRPV1	29421435
		lncRNA ENSMUST00000150952	Down	SDH	Mbp	30599267
		lncRNA AK081017	Down	SDH	Usp15	

AQP, amniotic aquaporins; BAI1, brain angiogenesis inhibitor 1; BDNF, brain-derived neurotrophic factor; CCI, chronic constriction injury; CDK, cyclin-dependent kinase; CELF2, CUGBP Elav-like family member 2; CSF, cerebrospinal fluid; DNP, diabetic neuropathic pain; DRG, dorsal root ganglion; ELAVL1, ELAV-like RNA-binding protein 1; ENO1, Alpha-enolase; EphB1, Eph receptor B1; EZH, Zeste homolog; HDAC4, histone deacetylase 4; HMGB1, high mobility group box-1; IRAK1, interleukin-1 receptor-associated kinases 1; JAG1, Jagged1; JAK, Janus kinase; LPAR3, lysophosphatidic acid receptor 3; MAPK, mitogen-activated protein kinases; NLRP3, NOD-like receptor family pyrin domain containing 3; NMDA, N-methyl-d-aspartate; Nrf2, nuclear factor erythroid 2-related factor 2; PDCD4, programmed cell death factor-4; PDK4, pyruvate dehydrogenase kinase 4; PNI, peripheral nerve injury; P2XR, P2X receptor; RAP1A, Ras-associated protein-1 A; SC, spinal cord; SDH, spinal cord dorsal horn; SNL, spinal nerve ligation; SNI, spared sciatic nerve injury; SN, sciatic nerve; STAT3, signal transducer and activator of transcription 3; TGF-β, Transforming growth factor-β; TRAF, TNF receptor associated factor; TRPV1, transient receptor potential vanilloid 1; ZEB1, Zinc finger E-box binding homeobox 1.

##### MicroRNAs

Peripheral nerve injury leading to NP is usually accompanied by miRNA alterations in DRG or other tissues, which has been proven to be associated with the severity of pain hypersensitivity (Adilakshmi et al., 2012; Chang et al., 2017; Wang Y. et al., 2021). MiRNAs bind to the 3′-untranslated region of target mRNAs, causing mRNA splicing and destabilization (Bartel, 2009). This is a crucial mechanism by which miRNAs are involved in NP. MiR-124-3p is downregulated in the DRG and SDH in a spared sciatic nerve injury (SNI)-induced NP rat model, leading to upregulation of Egr1 (Jiang et al., 2021). Analogously, miR-206-3p is decreased in the DRG of chronic constriction injury (CCI) rats and histone deacetylase 4 (HDAC4) is identified as a potential target of miR-206-3p. Intrathecal injection a lentivirus encoding miR-206-3p (LV-miR-206-3p mimic) upregulates the level of miR-206-3p in DRG of rat and alleviates NP induced by PNI (Wen et al., 2019). In addition, accumulative studies have shown that miR-223, miR-144, miR-140-3p, miR-122-5p, miR-30b-5p, and miR-340-5p, are highly decreased in the CCI model and verify their roles in the development of NP (Gao et al., 2019; Zhang X. et al., 2020; Cheng et al., 2021; Wan et al., 2021; Zhu et al., 2021; Liao et al., 2022). However, some miRNAs contributed to NP are contrary to the conventional trend. Upregulation of miR-30c-5p is observed in the DRG, spinal cord, plasma, and cerebrospinal fluid (CSF) of SNI rats, and is robustly consistent with the severity of allodynia (Tramullas et al., 2018).

Furthermore, the regulation of voltage-gated channels in DRGs by miRNAs has been considered another critical mechanism in CP in recent years. Following PNI-induced NP in rats, for instance, voltage-dependent sodium (Nav) 1.7 encoded by the SCN9A gene in the afferent fiber is proven to be involved in the development of NP (Xue et al., 2021). Interestingly, intrathecal administration of miR-30b agomir, chemically modified double-strand miR-30b mimic, shows an obvious decrease in Nav1.7 in DRG neurons and alleviates pain behaviors remarkably (Shao et al., 2016). The above evidence has indicated the regulatory effect of miRNAs on voltage-gated sodium channels in DRGs. In addition to miR-30b, upregulation of miR-137 suppresses the expression of voltage-gated potassium channel (Kv) Kv1.2 and increases mechanical allodynia and thermal hyperalgesia in CCI rats (Zhang J. et al., 2021). Downregulation of miR-137 restores the abnormal Kv currents and excitability in DRG neurons and thus attenuating NP. Moreover, voltage-gated calcium channels related to CP are also regulated by miRNA. MiR-34c-5p could bidirectionally regulate voltage-gated calcium (Cav) 1.2 channels, which proves that they can interact with each other to regulate hyperalgesia (Favereaux et al., 2011).

In addition to acting on voltage-gated channels, miRNAs also interact with numerous receptors in PNI-induced NP. Taking toll-like receptors (TLRs) as a common example, the expression of TLR8 is highly increased in IB4^+^ DRG neurons in a spinal nerve ligation (SNL) model, and inhibition of TLR8 alleviates SNL-induced pain (Zhang et al., 2018). Moreover, miRNAs interact with TLR8 highly associated with NP. Mechanistically, the miR-21-TLR8 signaling axis is identified as an important target in the SNL model (Zhang et al., 2018). TLR5 is also observed to be upregulated in bilateral CCI rats regulated by miR-217 (Jiang et al., 2019). Downregulation of the level of miR-217 exerts an inhibition effect of TLR5 through suppressing neuroinflammation and alleviates NP *in vivo* ultimately. Additionally, the C-X-C motif chemokine receptor (CXCR) is reported to be involved in the regulation of NP. Dong et al. (2021) have proved that the C-X-C motif chemokine ligand-12 (CXCL12)-CXCR4 pathway is regulated by miR-130a-5p in the development of NP. MiR-130a-5p mimics can attenuate CCI-induced NP *in vivo* by inhibiting the activation and inflammatory response of astrocytes, as well as inactivating CXCR4 and its downstream targets, such as Rac1 and nuclear factor-κB (NF-κB), etc. (Dong et al., 2021). Further study might be performed to explore more novel receptors contributing to the pathogenesis of PNI related to NP, providing for more strategies to treat NP.

The crosstalk between immune system and nervous system may be a novel pathogenic mechanism (Pinho-Ribeiro et al., 2017). It has been reported that exosomes and its cargos, including miRNAs, mediate the crucial crosstalk between different cells, organs, or systems, and are involved in the regulation of NP (Simeoli et al., 2017; D’Agnelli et al., 2020). Simeoli et al. (2017) have verified that exosomal miR-21-5p, as one of the cargos of sensory neuron-derived exosomes, is released after capsaicin activation of transient receptor potential vanilloid 1 (TRPV1) receptor in cultured DRG. *In vivo*, intrathecal injection of miR-21-5p antagomir, the inhibitor of miR-21-5p, attenuates neuropathic hypersensitivity and reduces the extent of inflammatory macrophage recruitment in the DRG. In brief, exosomal cargos play critical roles in sensory neuron–macrophage communication after damage to the peripheral nerve, and similar remote communication in the NP deserves further investigation.

##### Circular RNAs

Accumulative studies have also shown that circRNAs are significantly altered in the SDH in rats with NP. Bioinformatics analysis has shown that the expression levels of three circRNAs, circRNA_013779, circRNA_008008, and circRNA_003724, are increased more than 10 fold in the SDH of the CCI model compared with the control group (Cao et al., 2017), indicating that these three circRNAs are involved in the pathogenesis of NP. In addition, circRNA_008008 and circRNA_013779, as the two largest nodes in the circRNA-miRNA interaction network, can interact with eight miRNAs (Cao et al., 2017).

Mechanistically, current studies have found that circRNAs regulate pain by acting as miRNA sponges and through the circRNA-miRNA-downstream molecular axis. CIRCZNF609 acts as miRNA sponge to inhibit the expression of miR-22-3p, which alleviates mechanical allodynia and thermal hyperalgesia levels through regulation of the expression of Tumor Necrosis Factor-alpha (TNF-α), interleukin-1 (IL-1), and IL-6 in L4-L6 spinal cord tissue of CCI rats (Li et al., 2020). CircRNA.2837 attenuates nerve damage by regulating autophagy by targeting the miR-34 family (including miR-34a, miR-34b, and miR-34c) (Zhou et al., 2018). Besides, some other circRNAs are also involved in PNI through a similar mechanism, such as circ_0005075 as miR-151a-3p sponge in CCI rats (Zhang J. Y. et al., 2021), circ-Ankib1 as miR-423-5p, miR-485-5p, and miR-666-3p sponges in PNI (Mao et al., 2019) and ciRS-7 as the miR-135a-5p sponge in CCI rats (Cai et al., 2020).

In addition, circRNAs play roles in regulation of various pain-related signaling pathways. Several studies have performed Kyoto Encyclopedia of Genes and Genomes (KEGG) analysis, showing that the Hippo signaling pathway, MAPK signaling pathway, and endocytosis are the most key pathways related to NP (Li N. et al., 2013; Chen and Lu, 2017; Huang et al., 2017). CircRNAs alleviate or aggravate pain by controlling signaling pathways, ultimately affecting the associated inflammatory levels. For example, circ_0005075 affects the expression level of Notch2 by inhibiting the level of miR-151a-3p, eventually leading to changes in cyclooxygenase-2 (COX-2), IL-6, TNF-α, and other cytokines in the CCI model (Zhang J. Y. et al., 2021). Previous studies have demonstrated that Notch signaling is activated, mediating mechanical hyperalgesia induction and maintenance in a rat model of NP (Xie et al., 2015). Additionally, circRNA ZRANB1 mediates the Wnt5a/β-catenin signaling pathway to aggravate NP by acting on the miR-24-3p/LPAR3 axis (Wei et al., 2020). However, regulation of circRNAs in the complex signaling pathway network of NP is still very limited, and further studies are needed to elucidate the downstream pathways participating in the regulation of pain.

##### Long non-coding RNAs

Similar to miRNAs and circRNAs, lncRNAs also play roles in regulating the process of PNI. Since there is abundant evidence that includes lncRNAs in PNI, we mainly focus on the common downstream targets related to PNI regulated by lncRNAs in the latest findings. However, current research on lncRNA with its targets related to NP is not in-depth. Most current research mainly focuses on the mechanism of the lncRNA/miRNA/target axis. LncRNAs can directly bind to several targets, such as many receptors, ligands or enzyme, and participate in the pathogenesis of PNI-induced NP. Although receptors are of various types, they can be roughly divided into ion channel receptors and non-ion channel receptors. The typical ionic receptors are the P2X receptor and EPHB1-NMDA. The other targets mainly include CXCL, Janus kinase (JAK), brain-derived neurotrophic factor (BDNF), high mobility group box-1 (HMGB1), cyclin-dependent kinases (CDKs) and programmed cell death factor-4 (PDCD4), etc.

P2X receptors, members of an ATP-gated ion channels family, are widely involved in CP regulated by ncRNAs (Xiong et al., 2019). LncRNA MRAK009713 is markedly increased and interacts with P2X_3_ to enhance mechanical and thermal hyperalgesia in CCI rats (Li et al., 2017). LncRNA LOC100911498 plays a key role in the pathophysiological process of NP and LOC100911498 siRNA can block P2X_4_Rs-mediated p38MAPK activation and BDNF release, attenuating NP in rats (Tang et al., 2021). Additional evidence of ion channel receptors involved in PNI is that overexpression of lncRNA uc.153 in SC increases NP by targeting miR-182-5p and subsequent modulation of EphB1-NMDA receptors (Zhang C. et al., 2020).

On the other hand, some targets related to inflammation and the cell cycle are identified to be related to PNI-induced NP regulated by various lncRNAs. HMGB1, as a critical target in NP, is expressed in multiple cells (Andersson et al., 2002), and can promote neuroinflammation and NP in animal models (Wen et al., 2021). Diverse lncRNAs, such as lncRNA FIRRE, lncRNA NEAT1, and lncRNA MALAT1, can regulate HMGB1 and are involved in PNI-induced NP (Xia et al., 2018; Ma et al., 2020; Wen et al., 2021). Inhibition of lncRNA MALAT1 reverses the abnormal increasing expression level of HMGB1 caused by CCI surgery in rats, which attenuates the development of NP and neuroinflammation (Ma et al., 2020). The LncRNA MALAT1/miR-129-5p/HMGB1 axis is confirmed to play a critical role. Another study has reported that downregulation of lncRNA FIRRE suppresses the secretion of microglial cell-derived proinflammatory cytokines and reduces NP by also suppressing HMGB1 expression (Wen et al., 2021). Additionally, the lncRNA NEAT1/miR-381/HMGB1 axis highly contributes to PNI-induced NP (Xia et al., 2018).

Cyclin-dependent kinases, cell cycle-related proteins, have also been shown to be engaged in pain-related behavior (Yokoyama et al., 2020). The lncRNA PKIA-AS1 directly regulates the expression and function of CDK6 in SNL-induced NP, exerting an effect on maintaining neuroinflammation and NP (Hu et al., 2019). Inhibition of lncRNA PKIA-AS1 decreases the expression of CDK6 and alleviates SNL-induced NP (Hu et al., 2019). The lncRNA SNHG1 induces NP by directly regulating CDK4 levels (Zhang J. Y. et al., 2020). WNT5A, a member of the WNT family, is reported to be related to pain behaviors, and inflammatory responses and could sensibilize spinal nerves in rat models of NP (Simonetti and Kuner, 2020). The lncRNA CRNDE/miR-146a-5p/WNT5A axis plays a central role in rats with CCI-induced NP (Zhang Q. et al., 2021). CXCL has the normal physiological function of inducing directed chemotaxis of nearby reactive cells and is also believed to play a role in peripheral NP (Braga et al., 2020). Knockdown of LncRNA SNHG5 in the DRG of SNL mice alleviates NP and inhibits the activation of astrocytes and microglia by targeting the miR-154-5p/CXCL13 axis (Chen et al., 2020). The JAK-STAT pathway is an original signal transduction pathway shared by multiple cytokines (Dominguez et al., 2008; Wang et al., 2016a) and has been reported to be regulated by lncRNAs in PNI-induced NP. Downregulation of lncRNA DILC in the spinal cord attenuates NP through SOCS3-induced suppression of the JAK2/STAT3 pathway while increasing the viability of primary microglia, suppressing apoptosis and inhibiting the production of interleukin (IL)-6 and IL-1β in microglia (Liu Y. et al., 2020). STAT3 is also regulated by lncRNA XIST. Increasing the lncRNA XIST in the spinal cord can contribute to CCI-induced NP in rats by downregulating miR-544 and upregulating STAT3 (Jin et al., 2018). The lncRNA Linc00052/miR-448/JAK1 axis also plays a key role in SNL-induced NP (Wang L. et al., 2020). PDCD4, a translation factor that binds to eIF4A, is regulated by various lncRNAs under different pathological conditions. For instance, miR-155 regulate the inflammatory response through SOCS1–STAT3–PDCD4 in atherogenesis (Ye et al., 2016) and is recently found its concrete role in NP. Overexpression of lncRNA DGCR5 in the spinal cord attenuates NP by sponging miR-330-3p and targeting PDCD4 in CCI rats (Peng et al., 2019). Zeste homolog 2 (EZH2), a contributor to microglial activation and NP, can bind to lncRNAs. The LncRNA Lncenc1 contribute to NP by targeting EZH2 and downregulating the brain angiogenesis inhibitor 1 (BAI1) gene in mouse microglia (Zhang Z. et al., 2021). Furthermore, upregulation of lncRNA GAS5 in the spinal cord decreases pain behaviors by targeting miR-452-5p/CELF2 (Tian et al., 2020).

In addition to the receptors above, amniotic aquaporins (AQP), special channel proteins with roles in PNI-induced NP, has been reported recently. Knockdown of AQP9 promotes the development of NP, and further investigation have revealed that the lncRNA MALA T1/miR-154-5p/AQP9 axis acts as a novel target for NP (Wu J. et al., 2020). In summary, multiple lncRNAs can bind to the same receptor, while a single lncRNA can target numerous receptors in the development of PNI-induced NP. More research is needed to elucidate the huge network between lncRNAs and their receptors.

#### Peripheral neuropathic pain-associated with diabetic neuropathic pain

**FIGURE 3:**
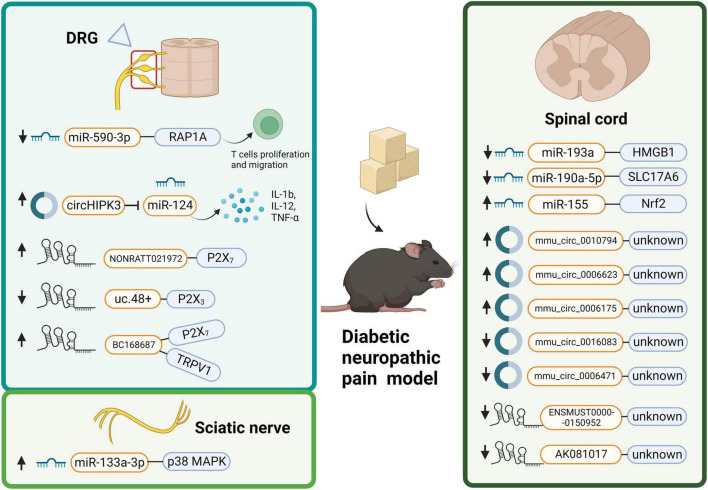
Distribution of dysregulated non-coding RNAs (ncRNAs) with the respective associated targets in diabetic neuropathic pain (DNP): ncRNAs (including microRNA, circular RNA, and long non-coding RNA) can participate in the development of DNP through various targets and mechanisms. NcRNAs displays different expressions in DRG, spinal cord and sciatic nerve of DNP models in DNP. DRG, dorsal root ganglion; HMGB1, high mobility group box-1; IL, interleukin; MAPK, mitogen-activated protein kinases; Nrf2, nuclear factor erythroid 2-related factor 2; RAP1A, Ras-associated protein-1 A; TNF, tumor necrosis factor; TRPV1, transient receptor potential vanilloid 1.

Diabetic neuropathic pain is a general complication of diabetes mellitus (DM), and patients with DNP suffer various degrees of pain (Feldman et al., 2017). Oral treatment is the most frequent topical treatment for DNP, but its use is often limited by systemic side effects (Yang et al., 2019). Since a large population suffers from DM worldwide with a high risk of DNP (Sloan et al., 2018), we must seek better treatment to alleviate DNP. Much evidence has shown that ncRNAs are dysregulated in DNP (Table 1) and have the potential to be a novel strategy (Figure 3).

##### MicroRNAs

A recent sequencing analysis has found that miRNAs are altered greatly in the spinal cord of a mouse model with streptozotocin (STZ)-induced DNP, while a total of 791 miRNAs are detected (He et al., 2021). Therein, 148 miRNAs, including 68 upregulated and 80 downregulated miRNAs, exhibit remarkably dysregulated expression 42 days after STZ injection in mice compared with those in the control group (He et al., 2021). KEGG signaling pathway analysis indicates that the “Rap1 signaling pathway,” “human T-lymphotropic virus-I infection,” and the “MAPK signaling pathway” are the top three pathways related to the differentially expressed miRNAs. Meanwhile, GO analysis in the category of the biological process shows that “multicellular organism development,” “developmental process,” and “regulation of cellular metabolic process” are the top three enriched processes among the differentially expressed miRNA target genes. Based on the research above, miRNAs exert potential functions in the development of DNP.

MicroRNAs affect diverse progress related to DNP, including inflammation-related cytokines, signaling pathways and interaction with various cells. For example, Yang et al. (2017) have revealed that the expression of miR-190a-5p is decreased and SLC17A6 is increased in the spinal tissue of the DNP model. In addition, reversing the expression of miR-190a-5p and SLC17A6 alleviates the nociceptive response and decreases IL-1β and IL-6 levels in DNP (Yang et al., 2017). Another study has demonstrated that miR-155 suppresses Nrf2 expression level (Chen et al., 2019). Silencing miR-155 alleviates SNI by promoting cell proliferation, inhibiting apoptosis, and decreasing inflammation in DNP (Chen et al., 2019). Also, miR-193a is decreased in the lumbar SDH of STZ-induced diabetic mice (Wu et al., 2019). Overexpression of miR-193a inhibits HMGB1 expression in the lumbar SDH accompanied by suppressing peripheral neuroinflammation to alleviate DNP (Wu et al., 2019).

In addition, miRNAs inhibiting or interfering with cell processes, such as migration, apoptosis, and infiltration may become one of the pathogenic mechanisms of DNP (Gumy et al., 2008; Wang et al., 2014; Feng et al., 2018). Regulation of these miRNAs to improve cell state will be a prospective idea in the treatments of DNP. T cells can migrate into the spinal cord in several pain models, exerting a pro-inflammatory effect, and immune cell infiltration is involved in DNP (Wu Y. et al., 2020). MiR-590-3p inhibit T cell infiltration to alleviate DNP (Wu Y. et al., 2020). Chang et al. (2020) have found that miR-133a-3p is upregulated in the sciatic nerves of DM rats. MiR-133a-3p mimics are transfected into RSC96 Schwann cells, increasing p-p38 MAPK levels in the sciatic nerve and contributing to the development of NP (Chang et al., 2020). In summary, miRNAs could be a new direction for the treatment and diagnosis of DNP and further explorations will be needed to reveal their application and extra functions in DNP.

##### Circular RNAs

He et al. (2021) have identified 2,118 distinct circRNAs in the spinal cord of STZ-induced DNP mice. Among them, 1,552 circRNAs are <1,000 nt, and the median length is 620 nt. Further analysis has indicated that the majority of circRNAs are derived from exons. Thereinto, mmu_circ_0010794, mmu_circ_0006623, and mmu_circ_0006175 are more significantly upregulated circRNAs, while mmu_circ_0016083 and mmu_circ_0006471 show the opposite trends (He et al., 2021). Specifically, Wang Z. et al. (2018) have explored the role of circHIPK3 in DNP. CircHIPK3 is abundant in serum of diabetes patients who suffer DNP and in DRG from STZ-induced DNP rats (Wang L. et al., 2018). Knockdown of circHIPK3 alleviates DNP by inhibiting IL-1b, IL-6, IL-12, and TNF-α protein expression in the DRG of diabetic rats. Furthermore, they have verified that circHIPK3 interacts with miR-124, decreasing its expression and that overexpression of miR-124 greatly decreases mechanical allodynia and thermal hyperalgesia *in vivo*. This research provides us with a novel pathway to treat DNP by interfering with the circHIPK3/miR-124 axis. Evidence above shows a strong connection between circRNAs and DNP. However, research on circRNAs and DNP is very limited, and further research is urgently needed.

##### Long non-coding RNAs

Long non-coding RNAs are involved in the processes of DNP. Clinical evidence that have enrolled 154 patients with DM type 2 reveals an upregulation of lncRNA NONRATT021972 associated with NP scores of DM type 2 (Yu et al., 2017). Animal experiments have demonstrated that lncRNA NONRATT021972 siRNA decreases inflammation by inhibiting TNF-α and attenuates NP in rats of STZ-induced DNP (Liu et al., 2016). Indeed, the expression of lncRNA NONRATT021972 is significantly higher in the DRG of the DM group than in the control group. Wang et al. (2016b) have found that lncRNA uc.48+ is downregulated in DM and further explored their roles in DNP. Upregulation of lncRNA uc.48+ can attenuate DNP by inhibiting the phosphorylation and activation of ERK1/2 and targeting the P2X_3_ receptor in DRG. In addition to the P2X_3_ receptor, P2X receptors have been widely identified in DNP (Wang et al., 2016b). P2X_7_ receptors, as ligand-gated ion channels, are verified in occurrence of CP in both pre-clinical and clinical research (Sorge et al., 2012; Hu et al., 2020) and recently find its role in regulation of DNP hypersensitivity (Liu et al., 2016; Wang A. et al., 2021). Knock-out of the P2X_7_ receptor decreases mechanical and thermal hypersensitivities in mice and activation of the P2X_7_ receptor shows an adverse effect (Zhao et al., 2016). Interestingly, both lncRNA BC168687 siRNA and lncRNA NONRATT021972 siRNA are identified to regulate DNP by targeting the P2X_7_ receptor in DRG (Liu et al., 2016, 2017). LncRNA BC168687 siRNA is also involved in TRPV1-mediated DNP in rats (Liu et al., 2018).

Genome-wide expression patterns of lncRNAs in the SDH of mice with DNP have shown that 1,481 lncRNAs are differentially expressed and 289 neighboring and 57 overlapping lncRNA-mRNA 15 pairs are identified (Du et al., 2019). Researchers have verified that lncRNA-mRNA, such as ENSMUST00000150952-Mbp and AK081017-Usp15, may play a critical role in DNP pathogenesis (Du et al., 2019). In summary, lncRNAs are widely altered in the occurrence of DNP, and more lncRNAs need to be elucidated as potential treatment targets for DNP patients in the future.

#### Central neuropathic pain associated with spinal cord injury

Spinal cord injury is a kind of general central nervous system trauma accompanied by a common complication: CNP as well as an abnormality of autonomic function (Eldahan and Rabchevsky, 2018; Viswanath et al., 2020). SCI patients with NP find it to be unbearable, and the long-term pain affects their sleep, work, and life (Siddall and Middleton, 2015). NcRNAs are involved in the development of CNP after SCI.

##### MicroRNAs

Multiple studies have focused on miRNA microarrays or genome-wide miRNA expression profiling screens to confirm their alterations after SCI-induced NP in clinical patients (Zhao et al., 2021), trying to find a new mechanism for this type of pain. A total of 2,367 distinct miRNAs in adults with pain after SCI are identified, and 71 miRNAs are differentially expressed in chronic NP (Ye et al., 2021). Among these, hsa-miR-19a-3p and hsa-miR-19b-3p are remarkably higher in chronic SCI with NP, and their potential clinical value for discriminating between patients with and without pain is verified (Ye et al., 2021). Moreover, miRNA-targeted therapy and its roles in SCI-induced CNP are increasingly studied. Yao et al. (2021) have elucidated that miR-130a-3p is upregulated in the spinal cord lesions of SCI rats. The inhibition of miR-130a-3p represses inflammatory cytokines, such as IL-1β, IL-6, and TNF-α, and upregulates the IGF-1/IGF-1R signaling pathway to alleviate NP caused by SCI (Yao et al., 2021). Upregulation of miR-139-5p improves locomotor functional recovery and attenuates both mechanical and thermal hypersensitivities, as well as promotes neuronal survival in the SCI model (Wang P. et al., 2021). These studies suggest that it would be beneficial to explore the mechanism of miRNAs as a strategy to treat pain following SCI.

##### Circular RNAs

There are very few studies of circRNAs directly involved in CNP in SCI models. However, circRNAs are proven to be associated with nerve cell inflammation, microglial activation, neuronal death, etc., after SCI (He et al., 2020; Tong et al., 2021). These pathophysiological processes are highly related to CNP, and circRNAs are verified for their direct role in CNP associated with SCI in further exploration. He et al. (2020) have observed that quietness circ 0000962 expression is downregulated in SCI model rats and overexpression of quietness circ 0000962 exerts an effect of inhibiting nerve cell inflammation in an *in vitro* model of SCI through activation of PI3K/Akt and suppression of NF-κB. Another study has found that circ-Usp10, as a competing endogenous RNA, could promote microglial activation and induce neuronal death by targeting miR-152-5p/CD84 in SCI (Tong et al., 2021). Additionally, circ-HIPK3 sponges miR-558 to inhibit neuronal cell apoptosis after SCI, exerting its neuroprotective effect (Wang L. et al., 2018). Although some of the diverse roles of circRNAs on neurons or microglia after SCI have been elucidated, whether their direct neuroprotective effects and possible mechanisms on sensory neurons in SCI deserve more original research.

##### Long non-coding RNAs

Spinal cord injury with CNP is a multifactorial pathological process, and lncRNAs have recently been regarded as promising biomarkers for CNP-associated SCI. Zhao et al. (2021) have detected the expression of lncRNAs and mRNAs in peripheral blood samples of patients who suffer from SCI with NP and without NP. Screening results have demonstrated that seven genes and two lncRNAs are directly involved in the pain pathway: E2F1, MAX, MITF, CTNNA1, ADORA2B, GRIK3, OXTR, LINC01119, and LINC02447 (Zhao et al., 2021). LINC01119 and LINC02447 have a positive correlation with these genes (Zhao et al., 2021). The expression of lncRNA NEAT1 is distinctly higher in rats with SCI with CNP (Xian et al., 2021). Mechanistically, overexpression of lncRNA NEAT1 mediates the expression of IL-6, IL-1β, and TNFα and further exploration have indicated that lncRNA NEAT1 is involved in SCI-induced NP progression through the lncRNA NEAT1/miR-128-3p/AQP4 axis (Xian et al., 2021). Another study has found that LncRNA PVT1 depletion significantly alleviates SCI with NP by inhibiting astrocytic activation and reducing the expression of neuroinflammatory factors (Zhang P. et al., 2021). The lncRNAPVT1/miR-186-5p/CXCL13/CXCR5 axis is regarded as a new therapeutic target for NP (Zhang P. et al., 2021). Therefore, it is critical to explore the interaction network between multiple lncRNAs and its targets.

#### Chronic regional pain syndrome

Complex regional pain syndrome is characterized by megalgia and inflammation, often affecting a single limb and following an injury (Kessler et al., 2020). It is difficult to meet a satisfactory analgesic treatment for CRPS. However, the pathogenesis of CRPS is still unclear. Here we focus on the role of ncRNAs in the development of CRPS, and almost all research concentrated on microRNAs in CRPS.

Circulating miRNAs, most encapsulated in small extracellular vehicles (sEVs) or exosomes, are stable in intercellular communication and may become an original biomarker or risk factor for CRPS (Ramanathan et al., 2019a; Wickman et al., 2021). Wickman et al. (2021) have purified and identified both cytokine and miRNA levels in serum sEVs in a tibia fracture model (TFM) induced-CRPS. The results have shown an unchanged level of cytokines but significantly differential expression of miRNAs. Thereinto, thirty sEV miRNAs are identified and correlated with CRPS clinical patients, which suggests a prospective prediction and diagnosis biomarker for CRPS. Another similar study has shown that miR-939 is robustly upregulation in sEVs from serum of CRPS patients (Ramanathan et al., 2019b). Levels of miR-939 in CRPS patients varies greatly in different immune cell-derived sEVs, especially higher in sEVs derived from B cells, T cells, and NK cells relative to monocytes in controls (Ramanathan et al., 2019b). We speculate that accurate detection of miRNAs in some related cell-derived sEVs is of great clinical significance and requires further research. In addition, Pande et al. (2021) have revealed 22-fold downregulation of the miRNA hsa-miR-605 in the blood of CRPS patients with poor analgesic effects of ketamine. Moreover, the level of serum hsa-miR-605 is highly correlated with CRPS, which regulates the proinflammatory chemokine CXCL5 and plays a critical role in leukocyte recruitment and activation (Pande et al., 2021).

Complex regional pain syndrome is more common in women than men (van Velzen et al., 2019). Interestingly, a recent study has confirmed that miRNAs and their roles can also lead to different gender incidences of CRPS. MiR-34a targets X inactivation-specific transcript (XIST) through the proinflammatory transcription factor Yin–Yang one under inflammation directly (Shenoda et al., 2018). XIST mediating X-chromosome inactivation causes a different incidence of CRPS in sex (Patrat et al., 2020). XIST is critical for dosage compensation in females, and thus downregulating XIST completely or directly could be an infeasible intervention strategy for female patients with CRPS (Shenoda et al., 2018). The findings above suggest that miRNA-mediated downregulation of XIST may be a potential method for pain relief in female patients. Although several studies have explored multiple mechanisms of miRNAs in CRPS, whether some miRNAs can be used as post-treatment efficacy evaluation of CRPS is still worthy of further study and exerts its broad prospects for diagnosis and therapeutic effect prediction.

#### Inflammatory pain

Inflammatory pain is a very common type of pain, especially in bone and joint diseases, such as osteoarthritis (OA), rheumatoid arthritis (RA), and multiple sclerosis (MS) (Muley et al., 2016; Conaghan et al., 2019; Mailhot et al., 2020). Clinically, we have very limited treatments for this severe type of pain. Recent evidence has shown that ncRNAs play an essential role in IP in both patients and rat models (Cheng et al., 2018; Pan et al., 2019).

##### MicroRNAs

MicroRNAs have proven to be promising targets for refractory IP (Descalzi et al., 2015). Exosomal miRNAs can mediate pain through communication between sensory neurons and immune cells (Simeoli et al., 2017). MiR-21 deletion in sensory neurons inhibits macrophage infiltration and attenuates pain in mice (Simeoli et al., 2017). This evidence indirectly indicates that miRNAs have a potential effect on pain-related immune response.

MiR-544-3p is markedly reduced in the DRG of RA mouse models and targets neuronal FcgRI to mediate acute joint pain hypersensitivity induced by IgG immune complexes (Liu et al., 2021). Similarly, miR-146a and the miR-183 cluster are also robustly downregulated in the DRG (L4/L5) and spinal cord after experiencing knee joint OA pain in rats (Li X. et al., 2013). Interestingly, miR-146a and the miR-183 cluster could mediate hyperalgesia by regulating diverse factors associated with inflammation in glial cells, such as IL-6, IL-1β, TNFα, NF-κB, TLR-2, and TLR-4. The miR-183 cluster can even affect pain-related ion channel genes (i.e., Nav1.3 and TRPV1) in microglial cells (Li X. et al., 2013). Besides, miR-485-5p (Xu et al., 2020), miR-16 (Chen et al., 2016), miR-211 (Sun et al., 2019), miR-451 (Sun and Zhang, 2018), and miR-34a (Liang et al., 2019) are decreased in IP animal models while miRNA-107 (Zhang L. et al., 2021) is proven to be upregulated in IP.

MicroRNAs can also regulate IP by participating in various signaling pathways. For instance, microRNA-211-5p enhances the analgesic effect of dexmedetomidine on TNBS-induced chronic inflammatory visceral pain in rats through the MEK/ERK/CREB pathway (Sun et al., 2019). Another study has observed that miR-216a-5p is downregulated and HMGB1 is upregulated in a complete Freund’s adjuvant-induced IP model (Zhenzhen et al., 2021). Furthermore, it is proven that miR-216a-5p targets the HMGB1-TLR4-NF-κB pathway and finally alters microglia-mediated neuroinflammation to alleviate inflammatory behavioral hypersensitivity (Zhenzhen et al., 2021). In summary, miRNAs can mediate IP or neuroinflammation through various mechanisms. We mainly review its role in interacting with downstream gene expression and molecules or targeting diverse signaling pathways.

##### Circular RNAs

Circular RNAs also play an essential role in IP. Pan et al. (2019) have found that circRNA-filip1L is notably increased in the ipsilateral SDH of mice with IP and that miRNA-1224 knockdown or Ago2 overexpression induces nociceptive behaviors in naive mice. Functional evidence has suggested that circRNA-Filip1l regulates IP by targeting Ubr5 in an Ago2-dependent manner (Pan et al., 2019). This study shows a novel epigenetic mechanism of interaction between miRNA and circRNA in chronic IP.

Circular RNAs can be used as biomarkers for many joint diseases. With similar mechanism as above, the role of the circRNF121/miR-665/MYD88 axis becomes a potential target for OA patients through the NF-κB pathway (Wang T. et al., 2020). Other evidence has shown that the levels of hsa_circ_0000175 and hsa_circ_0008410 in peripheral blood monocytes improve the diagnostic accuracy of rheumatoid joints and are associated with the activation, severity, and the amount of joint tenderness of RA (Luo et al., 2020b). Similarly, the expression level of hsa_circ_0079787 is related to the severity of axial spondylarthritis (Luo et al., 2020a). The evidence above has shown that circRNAs may be related to the severity of joint diseases or pain biomarkers. However, whether hsa_circ_0008410 are involved in RA-induced pain or not needs to be further studied.

Additionally, several studies have focused on the circRNAs involved in the pathogenesis of low back pain and intervertebral disc degeneration (Li et al., 2021a). Downregulation of circRNAs such as circRNA-CIDN, circSEMA4B, and circRNA GRB10 promotes low back pain by regulating apoptosis, signaling pathways, and sponging different miRNAs (Guo et al., 2018; Wang X. et al., 2018; Xiang et al., 2020). Circ-FAM169A may act as a competitive endogenous RNA to sponge miR-583 and regulate intervertebral disc degeneration pathological process (Li et al., 2021a). These studies have described circRNAs as novel diagnostic biomarkers of diseases and have showed effective strategies to alleviate the severity of disease and pain.

##### Long non-coding RNAs

Accumulated studies have indicated the essential role of lncRNAs in IP, such as OA and RA (Budd et al., 2018; Lü et al., 2020). In an experiment on the treatment of OA, Yang et al. (2021) have tried to determine the underlying mechanism of lncRNA H19 secreted by umbilical cord blood mesenchymal stem cells (UCBMSCs) in the treatment of advanced OA-induced pain. LncRNA H19 is enriched in the exosomes of UCBMSCs and lncRNA H19 affects pain and central sensitization of advanced OA through the microRNA-29a-3p/FOS axis (Yang et al., 2021). Another study has shown that the expression of lncRNA SNHG14 and IL-1β mRNA are both upregulated in OA. Downregulation of lncRNA SNHG14 enhances the proliferation of IL-1β-treated chondrocytes and inhibits cell apoptosis (Wang B. et al., 2021). Moreover, knocking down lncRNA SNHG14 decreases the progression of OA in rats by inhibiting inflammatory responses. Downregulation of lncRNA SNHG14 inhibits FSTL-1-mediated activation of NLRP3 and TLR4/NF-κB signaling pathway activation through miR-124-3p to alleviate inflammatory reactions in OA (Wang B. et al., 2021). However, whether lncRNA SNHG14 and its target has an effect on OA-induced pain needs further confirmation.

In addition to OA, researchers have established a rat model of neuroinflammatory pain by human immunodeficiency virus envelope glycoprotein 120 (gp120) treatment (Peng et al., 2021). Downregulation of lncRNA uc.48+ significantly alleviates hyperalgesia in gp120-treated rats (Peng et al., 2021). P2Y_12_ receptors are finally found to be a downstream molecular target for lncRNA uc.48+ in neurons and heterologous cells (Peng et al., 2021). LncRNA-mediated gene regulation occurs in primary sensory neurons of the DRG during RA-like joint inflammation or RA-induced pain (Bai et al., 2020). A lncRNA-mRNA microarray has shown that 69 lncRNAs (42 up and 27 down) are significantly altered in a rat model of joint inflammation (Bai et al., 2020). Thereinto, the remarkable upregulation lncRNA MRAK163594 and lncRNA uc.247+ are co-expressed with the pain-related gene Scn2a, which contributes to pain conditions, such as inflammatory joint pain (Bai et al., 2020). The evidence above provides us with numerous potential targets for the diagnosis or therapy of diverse IP.

#### Cancer-induced pain

Cancer-induced pain is caused by primary cancer itself or metastases with moderate to severe pain among 90% of individuals with advanced cancer, which is an increasingly prominent public health problem (Treede et al., 2019). The current therapeutic regimen for CIP could not meet all needs for analgesia. Clinically, bone cancer pain (BCP) is a common type of CIP. We mainly focus on the role of ncRNAs in BCP as well as other types of CIP.

##### MicroRNAs

Overexpression of miR-300 suppresses expression of its target HMGB1 in rat model of cancer-induced bone pain, which significantly attenuates BCP (Liu C. et al., 2020). In addition to BCP, Zhu et al. (2020) have found that miR-330 is highly expressed in the SDH of nude mice with pancreatic cancer pain and microinjection of miR-330 mimic induces abdominal mechanical hypersensitivity in normal nude mice. MiR-330 robustly suppresses the expression of GABABR2 in the SDH of nude mice with pancreatic cancer pain (Zhu et al., 2020).

Alterations in gene expression, such as miRNAs, play a crucial role and affect the degree of pain. Bali et al. (2013) have found that 57 miRNAs are dysregulated in sensory neurons corresponding to tumor-related areas with genome-wide miRNA screening. Among these, miR-1a-3p could target Clcn3, an important gene encoding the chloride channel in CIP (Bali et al., 2013). Knocking down Clcn3 expression with Clcn3-siRNA enhances tumor-induced mechanical allodynia. Similarly, the effect of miRNA on Cav also plays an important role in the pathogenesis of CIP. For instance, knocking down the expression of Cav2.3 specifically in DRG neurons leads to hypersensitivity in mice (Gandla et al., 2017). Besides, miR-34c-5p and Cav2.3 are co-expressed both in cultured sensory neurons and DRG in mice with CIP. Researchers have concluded that miR-34c-5p target Cav2.3, exerting a pro-nociceptive effect (Gandla et al., 2017). Above all, miR-34c-5p might be a promising strategy to reach a satisfactory effect in further clinical therapy. Moreover, miRNAs can also affect synapses related to algesia conduction. MiR-124 is a specific inhibitor of synaptopodin (Synpo), a key protein for synaptic transmission (Elramah et al., 2017). MiR-124 is decreased and Synpo is increased in the spinal cord under CIP conditions. Elramah et al. (2017) have found that Synpo acts as a crucial component in nociceptive pathways. Intrathecal administration of miR-124 mimics in mice with BCP decreases Synpo expression and completely alleviates CIP in the early phase of cancer (Elramah et al., 2017). In conclusion, the evidence above provides us with broad insight into seeking better analgesia therapy plans for patients who suffer CIP.

##### Circular RNAs

Limited studies have shown that circRNAs have a potential effect on the pathogenesis of CIP. Chen et al. (2021) have validated eight differentially expressed circRNAs in the rat spinal cord by agarose gel electrophoresis and Sanger sequencing. CircSlc7a11 is selected for further functional study. Overexpression of circSlc7a11 greatly promotes cell proliferation and represses apoptosis of LLC-WRC 256 and UMR-106 cells (Chen et al., 2021). Besides, several signaling pathways, such as the chemokine signaling pathway, are verified to be linked to changes in circSlc7a11. Several potential targets of circSlc7a11, such as Pax8, Isg15, and CxCL10, and a complex circRNA/miRNA/mRNA network are involved in BCP (Chen et al., 2021). The effect of circSlc7a11 on the pathogenesis of BCP could be found through multiple pathways such as tumor cell invasion and proliferation, expression of multiple miRNAs and functional genes, etc. Screening differentially expressed circRNAs in BCP shows that 850 are obviously upregulated whereas 644 are significantly downregulated in the BCP group (Zhang Y. et al., 2020). Specifically focused on the role of circStrn3 in BCP, knockdown of circStrn3 can promote bone cancer cell apoptosis and proliferation (Zhang Y. et al., 2020). CircStrn3 regulates rno-miR-9a-5p to target Nfkb1, and Nfkb1 is found to be positively expressed in BCP rats and associated with the NF-κB pathway, indicating that circStrn3 might be a potential biomarker for BCP (Zhang Y. et al., 2020).

##### Long non-coding RNAs

Many lncRNAs have been verified to play a crucial role in cancer or cancer metastasis. However, we have ignored the functions of lncRNAs in CIP before and an increasing number of studies have focused on lncRNAs related to the occurrence of CIP. Microarray analysis has identified the differentially expressed lncRNAs and mRNAs in the DRG 14-days post-operation in the BCP rat model (Wei et al., 2021). A total of 73 lncRNAs and 187 mRNAs are dysregulated. The top 30 lncRNA-predicted mRNAs are mainly related to peroxisome, double-stranded RNA-binding, DNA-dependent DNA replication, tuberculosis, and purine metabolism (Wei et al., 2021). These findings suggest a strong correlation between lncRNAs and CIP.

Interestingly, upregulation of lncRNA-NONRATT021203.2 in the DRG contributes to cancer-induced hyperalgesia (Sun et al., 2020). In the underlying mechanism, CXCL9, mainly expressed in CGRP-positive and IB4-positive DRG neurons, is found to act as a target of lncRNA-NONRATT021203.2 by colocalizing with lncRNA-NONRATT021203.2 in DRG neurons (Sun et al., 2020). Moreover, lncRNA-NONRATT009773.2 is also identified to be involved in BCP. Depletion of lncRNA NONRATT009773.2 alleviates BCP and further lncRNA NONRATT009773.2/miR-708-5p/CXCL13 axis is confirmed to be engaged in the initiation and maintenance of neuroinflammation and hyperalgesia of BCP (Chen et al., 2022). The findings above indicate that the lncRNA/miRNA axis will be a novel strategy for the treatment of CIP.

## Conclusion and future perspectives

In summary, CP is a major health problem affecting the quality of life because of the lack of understanding of the underlying pathophysiology and effective and safe treatments. CP is a multifactorial disease and multiple mechanisms are involved in its pathogenesis (Cohen et al., 2021). For example, low socioeconomic status, poor working conditions, unstable home life, low education levels, and living in deprived environments are associated with increased pain (van Hecke et al., 2013). NcRNAs are one of the factors that can affect CP and have been studied extensively and are considered to be novel biomarkers and pharmacological applications in CP (Lötsch et al., 2013; Bali and Kuner, 2014; Lopez-Gonzalez et al., 2017). However, there are some limitations in the field of ncRNAs and CP. Most existing studies are confined to the ncRNA/receptor axis or RNA sequencing in DRG or spinal cord tissue in rodent animal models of CP. MiRNAs are the most well studied among the family of ncRNAs, their presence at the forefront of ncRNA-based CP therapeutics is not yet satisfied, and it is difficult to definitively categorize the majority of miRNAs as neither suppressors nor promoters of CP. Additionally, it is still unclear how ncRNAs directly bind to the target and the connection of diverse complex ncRNAs in different CP conditions, which deserves further investigation. Recent technological advancements might not allow a comprehensive assessment of their functional roles in multiple types of CP.

Although ncRNAs are promising for clinical applications in CP, there are still many challenges we face. Concrete issues must be considered, such as the quality, stability, safety of ncRNAs, and even biological activity until reaching the target location. Regarding the quality and stability of ncRNAs, degradation by ubiquitous RNase is an unneglectable problem if ncRNAs are considered to be pharmaceuticals for CP patients. More technological advances are needed to synthesize and manufacture multiple standard ncRNA mimics and inhibitors for clinical use in the future. Secondly, whether exogenous administration of ncRNAs for clinical use will produce unpredictable immune responses is a tricky problem we need to evaluate in advance. In addition, off-target effects are the main reason for side effects and poor healing efficacy in miRNA-based therapy (Jackson and Linsley, 2010). The properties of ncRNAs, including high molecular weight and hydrophilia determine the difficulties of traversing biological membranes if their targets are intracellular or intranuclear. Therefore, ncRNA-based therapeutics require a reliable delivery system across the membrane obstacle and protection from degradation and clearance, such as exosomes, liposomes, lentivirus, etc., as a delivery system (Vickers et al., 2011; El Andaloussi et al., 2013). Additionally, existing studies are limited to cell or animal models, and more pre-clinical studies and clinical trials are needed to verify the potential therapeutic effects of ncRNAs in CP. It is still a challenge to apply ncRNA-based strategies for diagnosis and therapeutics to the human body.

## Author contributions

CZ and RG mainly contributed to designing and writing the manuscript. RZ, HC, and CL retrieved literature. TZ provided administrative support. CC critically revised the manuscript. All authors wrote the article and read and approved the final manuscript.

## Abbreviations

AQP: amniotic aquaporins
BAI1: brain angiogenesis inhibitor 1
BCP: bone cancer pain
BDNF: brain-derived neurotrophic factor
Cav: voltage-gated calcium
CCI: chronic constriction injury
CDKs: cyclin-dependent kinases
CELF2: CUGBP Elav-like family member 2
CIP: cancer-induced pain
circRNAs: circular RNAs
CNP: central neuropathic pain
CP: chronic pain
CRPS: complex regional pain syndrome
CSF: cerebrospinal fluid
CXCR: C-X-C motif chemokine receptor
COX: cyclooxygenase
DM: diabetes mellitus
DNP: diabetic neuropathic pain
DRG: dorsal root ganglion
gp120: glycoprotein 120
ELAVL1: ELAV-like RNA-binding protein 1
ENO1: Alpha-enolase
EphB1: Eph receptor B1
EZH: Zeste homolog
HDAC: histone deacetylase
HMGB-1: high mobility group box-1
IL: interleukin
IP: inflammatory pain
IRAK1: interleukin-1 receptor-associated kinases 1
JAG1: Jagged1
JAK: Janus kinase
KEGG: Kyoto Encyclopedia of Genes and Genomes
Kv: voltage-gated potassium
LPAR3: lysophosphatidic acid receptor 3
lncRNAs: long non-coding RNAs
MAPK: mitogen-activated protein kinases
miRNAs: microRNAs
mRNA: messenger RNA
MS: multiple sclerosis
Nav: voltage-dependent sodium
ncRNAs: non-coding RNAs
NF-κB: nuclear factor-κB
NLRP3: NOD-like receptor family pyrin domain containing 3
NMDA: N-methyl-d-aspartate
NP: neuropathic pain
Nrf2: nuclear factor erythroid 2-related factor 2
OA: osteoarthritis
PDCD4: programmed cell death factor-4
PDK4: pyruvate dehydrogenase kinase 4
PNI: peripheral nerve injury
P2XR: P2X receptor
RA: rheumatoid arthritis
RAP1A: Ras-associated protein-1 A
RISC: RNA-induced silencing complex
SC: spinal cord
SCI: spinal cord injury
SDH: spinal cord dorsal horn
sEVs: small extracellular vesicles
SN: sciatic nerve
SNI: spared sciatic nerve injury
SNL: spinal nerve ligation
STAT3: signal transducer and activator of transcription 3
STZ: streptozotocin
Synpo: synaptopodin
TGF-β: transforming growth factor-β
TLRs: toll-like receptors
TNF: tumor necrosis factor
TRAF: TNF receptor associated factor
TRBP: transactivation response RNA-binding protein
TRPV1: transient receptor potential vanilloid 1
UCBMSCs: umbilical cord blood mesenchymal stem cells
XIST: X inactivation-specific transcript
ZEB1: Zinc finger E-box binding homeobox 1.
